# Glass Transition in Crosslinked Nanocomposite Scaffolds of Gelatin/Chitosan/Hydroxyapatite

**DOI:** 10.3390/polym11040642

**Published:** 2019-04-09

**Authors:** Karina N. Catalan, Tomas P. Corrales, Juan C. Forero, Christian P. Romero, Cristian A. Acevedo

**Affiliations:** 1 Departamento de Física, Universidad Técnica Federico Santa María, Valparaíso 2340000, Chile; kcatalans@gmail.com (K.N.C.); tomas.corrales@usm.cl (T.P.C.); christian.romero@usm.cl (C.P.R.); 2 Centro de Biotecnología, Universidad Técnica Federico Santa María, Valparaíso 2340000, Chile; jcarlosforero@gmail.com

**Keywords:** crosslinking, glass transition, nanoparticles, polymers, scaffolds

## Abstract

The development of biopolymeric scaffolds crosslinked with nanoparticles is an emerging field. Gelatin/chitosan scaffolds are gaining interest in medical areas, e.g., bone tissue engineering, given their suitability for nano-hydroxyapatite incorporation. The glass transition temperature is a thermodynamic property of polymer scaffolds that changes with crosslinker or nanofiller concentration. Here, we report the experimental change in glass transition temperature of gelatin/chitosan scaffolds modified by hydroxyapatite nanoparticles and crosslinker concentration. Our results show synergic effects between nanoparticles and crosslinking, which leads to a non-linear behavior of the glass transition temperature. Furthermore, a theoretical model to predict glass transition is proposed. This model can be used as a mathematical tool for the design of future scaffolds used in bone tissue engineering.

## 1. Introduction

In recent years, the development of biopolymeric scaffolds infiltrated with nanoparticles, i.e., nanocomposite scaffolds, have gained increasing interest for their applications in tissue engineering [1]. The benefits and toxic effects of nanoparticle addition within scaffolds have been extensively reported in literature, however, the thermodynamic aspects of their addition have not been studied in detail. Paul and Robeson reviewed the effects of nanofillers, such as clay-based nanocomposites, on the glass transition temperature ($T_{g}$) of a polymer matrix [2]. The glass transition temperature characterizes polymers, copolymers and biopolymers [3], as well as polymer-based composites. The $T_{g}$ of nanocomposites with well-dispersed nanofillers can exhibit considerable deviations relative to the equivalent bulk polymer value. In some cases, $T_{g}$ decreases as the interface between polymer and nanofiller produces free surfaces, and increases when the wetted interface yields attractive interactions [4]. Furthermore, biopolymer scaffolds used for tissue engineering must be crosslinked to avoid the dissolution in water. It is well known that the $T_{g}$ of scaffolds increases significantly with crosslinking [5]. The change of glass transition temperature can be correlated with important aspects of the polymer structure, both micro- and nanostructure, which in turn affects the behavior of cells seeded on the tissue engineered scaffolds. Important microstructure properties and nanostructure characteristics can be explained and modeled by means of polymer theory, in particular their glass transition temperature ($T_{g}$) [6,7,8].

Biopolymer scaffolds made with mixtures of gelatin and chitosan are gaining interest in medical areas such as tissue engineering. Furthermore, gelatin/chitosan scaffolds are being used as suitable materials for the incorporation of nanofillers, like hydroxyapatite, which is used in bone tissue engineering [9].

The understanding of glass transition behavior in crosslinked nanocomposite scaffolds is very important for the design of these materials. Glass transition studies have been used to design skin [10] and bone scaffolds [9], to understand the biodegradation of scaffolds [11] and to study the effect of sterilization using gamma radiation [12]. Reported values of $T_{g}$ for bone tissue engineering scaffolds are in the range of 30–40 ${}^{{}^{\circ}}$C [9]. However, to the best of our knowledge, there are no readily available studies reporting the design of scaffolds as a function of both nanoparticle and crosslinker concentration simultaneously. In this work, we report changes in the glass transition temperature $T_{g}$ of crosslinked scaffolds used for bone tissue engineering made of gelatin/chitosan/hydroxyapatite. We have analyzed these changes as a function of crosslinking and nanoparticle concentration, and propose a model that can be used as a scaffold design tool.

## 2. Materials and Methods

The crosslinked nanocomposite scaffolds were prepared using the freeze-drying method reported by Forero et al. [9], to prepare bone tissue engineering biomaterials. In brief, solutions of chitosan (0.5% w/v; 120 kDa; >85% deacetylated; Quitoquimica, Concepcion, Chile) and gelatin (0.25% w/v; type B; Merck, Darmstadt, Germany) were prepared at 50 ${}^{{}^{\circ}}$C in diluted acetic acid (100 mM). The solutions were modified by incorporation of hydroxyapatite nanoparticles (<200 nm; Sigma-Aldrich, St. Louis, MO, USA) and glutaraldehyde as crosslinker (Sigma-Aldrich, St. Louis, MO, USA). The solutions were poured onto Petri dishes, frozen at −20 ${}^{{}^{\circ}}$C and freeze-dried using the method described by the same authors [9].

To study the effects on $T_{g}$ by nanoparticles and crosslinking, an experimental design was carried out. In this design two factors are included (full factorial design $3^{2}$): Nanoparticle mass fraction (*X*) and crosslinker concentration (*C*). Each of these two parameters had three different levels. The levels for *X* were 0 (no nanoparticle added), 0.07 and 0.14. The levels for *C* were 0.02%, 0.06% and 0.10%. Statistical analyses were performed using the Modde software (Umetrics, Umea, Sweden).

The glass transition temperatures of the scaffolds were determined with a differential scanning calorimeter (Mettler Toledo, model DSC1, Greifensee, Switzerland), using the method previously described by Acevedo et al. [13].

## 3. Results and Discussion

### 3.1. Glass Transition Behavior

Glass transition is a key variable to design polymer scaffolds for tissue engineering. Given that $T_{g}$ changes when the scaffold is crosslinked [8], irradiated [12] or biodegradated [11], it allows the detection of variations in the fabrication, sterilization or storage of the scaffold. Also, the $T_{g}$ can be related with the nanostructure, which in turn, directly affects the cell behavior [6,7,8].

**Figure 1:**
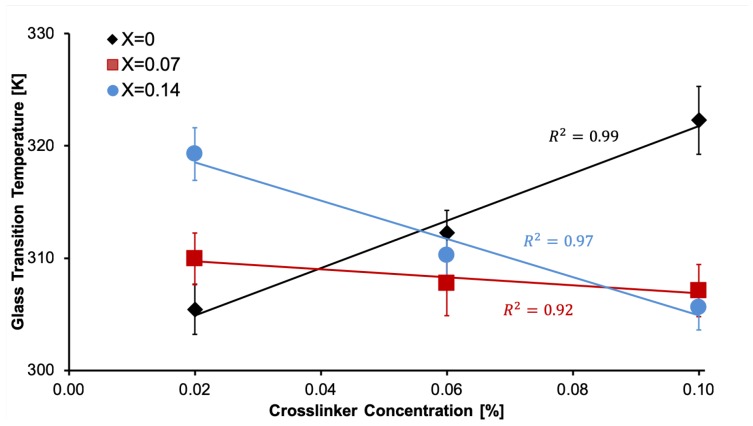
Experimental data of $T_{g}$ in crosslinked nanocomposite scaffolds. Three mass fractions of nanoparticles were measured: X=0, X=0.07 and X=0.14.

Figure 1 shows the results of the glass transition temperature. When no nanoparticles are added (X=0), the $T_{g}$ increases with the crosslinker concentration. This is a well-known phenomenon, that is due to the increase in the molecular weight of the polymer and can be described using the empirical Flory–Fox Equation [14]. However, when nanoparticles are used (X=0.07 and X=0.14), $T_{g}$ decreases as shown in Figure 1. It has been previously informed in nanocomposites with dispersed nanofillers [12,13,14], that $T_{g}$ can increase or decrease depending of the interaction that occurs within the material.

**Figure 2:**
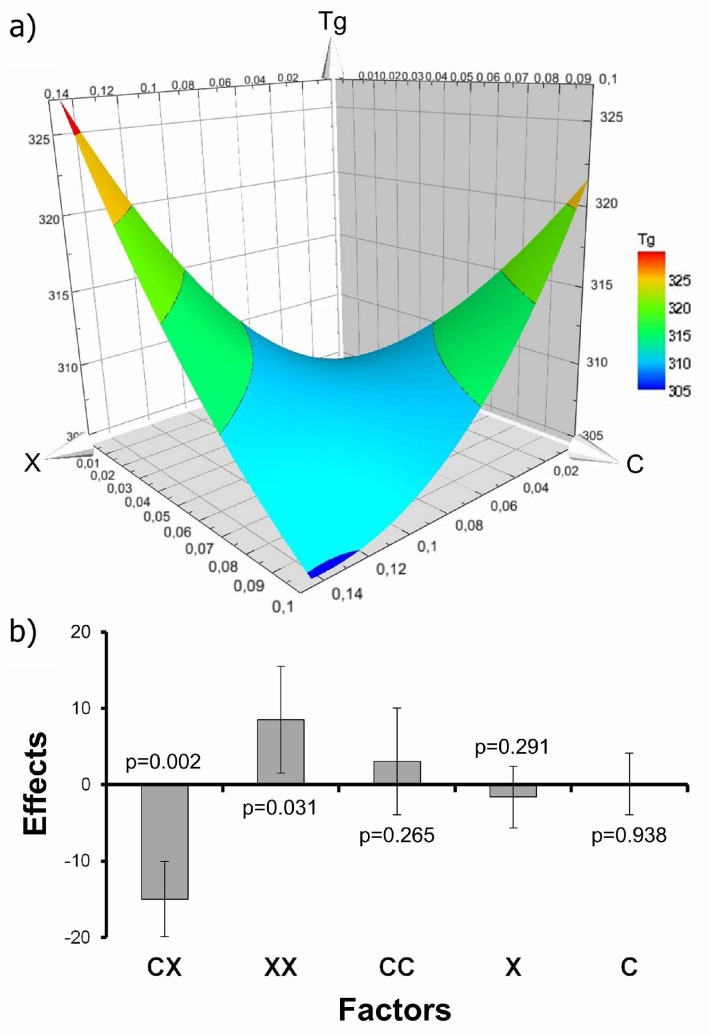
Results of the experimental design analysis for glass transition. (a) Surface plot for $T_{g}$ as a function of *C* and *X*. (b) Effects plots (with *p*-values and error bars 95%) for $T_{g}$.

In Figure 2 we show the results of the experiment design analysis. Figure 2a shows that the effects of *C* and *X* on $T_{g}$ are non-linear and that there are possible synergic effects between them. In Figure 2b we show that there is a nanoparticle-crosslinker interaction (CX) and a quadratic dependence on *X*. The estimation of the glass transition can be expressed with the equation $T_{g}\left(C,X\right)=k_{1}+k_{2}CX+k_{3}X^{2}$ (where $k_{i}$ are empirical parameters). Given that this model is empirical, it cannot be easily extrapolated to other systems. To further understand our experimental results, it is necessary to derive a theoretical model that can be extended to different scaffolds. This will enable a description of the interactions between nanoparticles *X* and crosslinks *C*, as well as the quadratic dependence on *X*.

### 3.2. Theoretical Modelling

There are several mathematical models to study $T_{g}$ in polymeric systems, e.g., thin films [15,16,17,18] and nanocomposites [19,20,21], but to the best of our knowledge, there are no available models to study $T_{g}$ in scaffolds as a function of both nanoparticle *X* and crosslinker concentration *C*.

The theoretical work was done to understand the change in $T_{g}$ observed in the scaffolds. To do this, we start with the model proposed by Lee et al. [19]. This model relates the glass transition with nanoparticle amount. We have modified this model to include the interaction between crosslink and nanoparticles. It has been reported [20], that the glass state formation is a result of the system’s loss of configurational entropy (*S*), which is given by the configurational entropies of the liquid ($S_{liquid}$) and glass states ($S_{glass}$), i.e., $S=S_{liquid}-S_{glass}$. In addition, we also follow Chow’s assumptions [21] that configurational entropies of glass state are zero and $\Delta C_{P}$, i.e., difference in heat capacity between the liquid and glass, is independent of temperature and composition, thus we can express the change in $T_{g}$ when nanoparticles (*X*) are added by [19,20,21]:

(1) $$\ln\left(\frac{T_{g}\left(X\right)}{T_{g}\left(X=0\right)}\right)=-\frac{1}{\Delta C_{P}}\left(S\left(X\right)-S\left(X=0\right)\right).$$

We assume that configurational entropies are composed by the disorientation entropies of polymer 1 ($S_{dis-1}$) and polymer 2 ($S_{dis-2}$), the mixing entropies ($S_{mix-12}$, $S_{mix-1X}$, $S_{mix-2X}$), the specific interaction entropies ($S_{spe-12}$, $S_{spe-1X}$, $S_{spe-2X}$) and the confinement entropies of the nanoparticles ($S_{con-1X}$, $S_{con-2X}$). The configurational entropies for $S\left(X\right)$ and $S\left(X=0\right)$ are given by:

(2) $$\begin{array}{cc} S\left(X\right)= & \;S_{dis-1}+S_{dis-2}+S_{mix-12}+S_{mix-1X}+S_{mix-2X}+S_{spe-12}+S_{spe-1X}+S_{spe-2X}\\ & +S_{con-1X}+S_{con-2X} \end{array}$$

and

(3) $$S\left(X=0\right)=S_{dis-1}+S_{dis-2}+S_{mix-12}+S_{spe-12}.$$

Then, by substituting these entropies in the above equation we obtain:

(4) $$\ln\left(\frac{T_{g}\left(X\right)}{T_{g}\left(X=0\right)}\right)=-\frac{1}{\Delta C_{P}}\left(S_{mix-1X}+S_{mix-2X}+S_{spe-1X}+S_{spe-2X}+S_{con-1X}+S_{con-2X}\right).$$

The expressions that relate each type of single entropy are described in Table 1 [19]. In addition, we will assume: (a) The specific interaction between polymers and nanoparticles are related linearly with the crosslinker concentration $\left(\gamma_{spe-iX}=\gamma_{ia}+\gamma_{ib}C\right)$, and (b) the volumetric fraction of nanoparticles is proportional to the mass fraction $\left(\phi_{X}\propto X\right)$. Then, by substituting the terms in Table 1 and factoring, we obtain:

(5) $$\ln\left(\frac{T_{g}\left(C,X\right)}{T_{g}\left(C,X=0\right)}\right)=\alpha_{1}+\alpha_{2}C+\beta_{1}X\ln X+\beta_{2}CX\ln X+\beta_{3}X^{2}+\alpha_{3}X^{3},$$

where $\alpha_{j}$ and $\beta_{j}$ (with j=1,2,3) are parameters obtained from regrouping other terms. By setting the boundary condition $\lim_{X\to 0}\ln\left(\frac{T_{g}\left(C,X\right)}{T_{g}\left(C,X=0\right)}\right)=0$, we obtain that: $\alpha_{1}+\alpha_{2}C=0$ $\left(\forall \;C\right)$. In addition, in order to simplify the equation, the higher order term $X^{3}$ is disregarded $\left(0\leq X<1\Rightarrow X^{3}\ll 1\right)$. With these considerations taken into account, we obtain a general expression for the change in the glass transition temperature:

(6) $$\ln\left(\frac{T_{g}\left(C,X\right)}{T_{g}\left(C,X=0\right)}\right)=\left(\beta_{1}+\beta_{2}C\right)X\ln X+\beta_{3}X^{2},$$

where $\beta_{1}$, $\beta_{2}$ and $\beta_{3}$ are parameters of the material that can be fitted to experimental data, or obtained if the molecular properties are known:

(7) $$\beta_{1}=\frac{k_{B}}{r_{X}\Delta C_{P}}\left(4-\left(\gamma_{1a}+\gamma_{2a}\right)\ln\left(\frac{z-1}{e}\right)\right),$$

(8) $$\beta_{2}=-\frac{k_{B}}{r_{X}\Delta C_{P}}\left(\left(\gamma_{1b}+\gamma_{2b}\right)\ln\left(\frac{z-1}{e}\right)\right),$$

(9) $$\beta_{3}=-\frac{4k_{B}}{r_{X}\Delta C_{P}}\left(\frac{\tanh\left(1-\frac{R_{X}}{r_{0,1}}\right)}{\phi_{1}}+\frac{\tanh\left(1-\frac{R_{X}}{r_{0,2}}\right)}{\phi_{2}}\right).$$

### 3.3. Experimental Application of the Model

**Figure 3:**
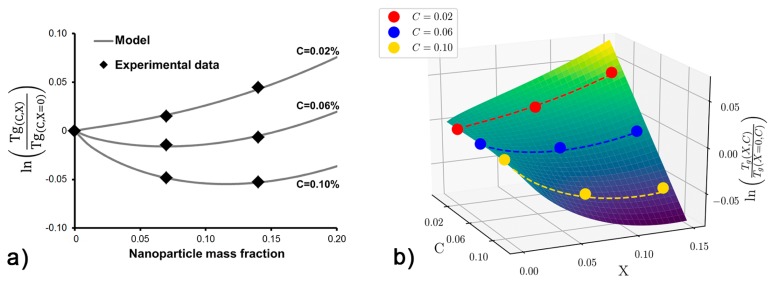
(**a**) Change in glass transition temperatures as a function of nanoparticles mass fraction for three different crosslinker concentrations. Solid lines represent our proposed model. (**b**) Reconstructed surface plot obtained using the fitted parameters ($\beta_{i}$) and experimental points (solid dots).

We apply Equation (6) to our experimental glass transition measurements and obtain intrinsic scaffold parameters ($\beta_{i}$). The data obtained of the full factorial design $3^{2}$ was used to contrast this model with experimental results (Figure 3).

Figure 3a displays a single set of fitting parameters for experimental information obtained previously. The obtained coefficient of determination was $R^{2}=0.999$. The experimental data is good agreement with the proposed model. The intrinsic material parameters $\beta_{1}$, $\beta_{2}$ and $\beta_{3}$ obtained from fitting the experimental data: $\beta_{1}=-0.136$, $\beta_{2}=4.359\left[{\%}^{-1}\right]$ and $\beta_{3}=1.506$.

Figure 3b shows the experimental data points together with the fitted surface obtained from the single set of fitted parameters: $\beta_{1}$, $\beta_{2}$ and $\beta_{3}$. The trends in the model show good agreement with the experimental results.

**Figure 4:**
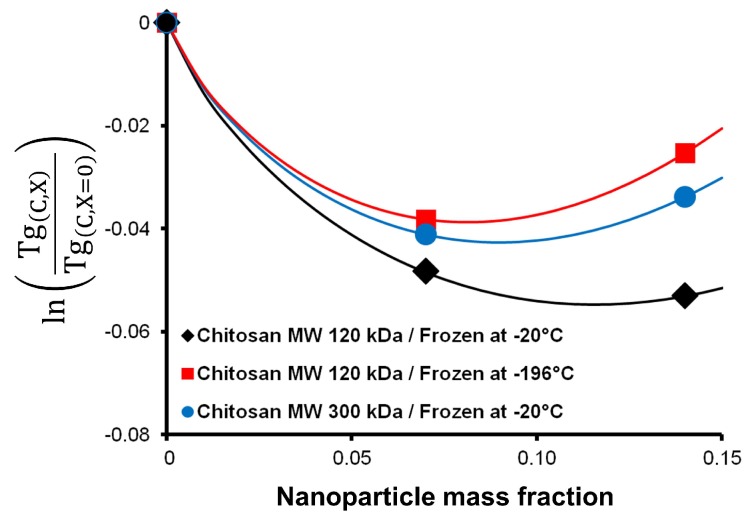
Application of the model to different systems using a fixed crosslinker concentration of 0.10%. Experimental data of the original scaffold (black diamonds), modification of the freezing temperature (red squares) and change of the chitosan molecular weight (blue dots) are shown.

We also tested the model on other systems, where we have modified the original scaffolds with a 0.10% crosslinker concentration. One modification was to change the freezing temperature, i.e., the previous step to freeze-drying, from −20 ${}^{{}^{\circ}}$C to −196 ${}^{{}^{\circ}}$C by using liquid nitrogen. The other modification was to change the molecular weight (MW) of the chitosan used in the scaffold from 120 kDa to 300 kDa. Figure 4 shows that molecular weight has a smaller effect on the shape of the curve than the freezing temperature. Overall, the scaffolds fabricated exhibit a similar behaviour in $T_{g}$, first decreasing and then increasing as a function of nanoparticle mass fraction.

Changes in $T_{g}$ were mathematically modelled, and predicted values come to good agreement with the experimental behavior. We propose that this model can be used in the design of crosslinking processes used in the fabrication of nanocomposite scaffolds. This is a first approach and more experimental work is undoubtedly necessary to validate the equation with other nanocomposite scaffolds.

## 4. Conclusions

The glass transition temperature of a biopolymer scaffold changes when it is crosslinked or modified by nanoparticles addition. Our results suggest that there are synergic effects between nanoparticles and crosslinking, which lead to a non-linear behavior of the glass transition temperature. Moreover, a theoretical model to estimate the $T_{g}$ was developed and fitted to experimental data. The model represents the experimental behavior of the glass transition and can be used as a mathematical tool for the design of scaffolds with specific physical properties, in particular $T_{g}$.

## Figures and Tables

**Table 1:** Configurational entropies used in the theoretical modelling, where $k_{B}$ is the Boltzmann constant, $\phi_{i}$ is the volume fraction of polymer i (with i=1,2), $\phi_{X}$ is the volume fraction of nanoparticles, $r_{i}$ is the molar volume of polymer *i*, $r_{X}$ is the molar volume of nanoparticles, $R_{X}$ is the nanoparticle radius, $r_{0,i}$ is the average monomer radius of the polymer *i*, *z* is the lattice coordination number and $\gamma_{spe-iX}$ is a parameter representing the specific interaction between the polymer *i* and nanoparticle.

Configurational Entropy	Equation
Mixing entropy	$S_{mix-iX}=-k_{B}\left(\frac{\phi_{i}}{r_{i}}\ln\phi_{i}+\frac{\phi_{X}}{r_{X}}\ln\phi_{X}\right)$
Specific interaction entropy	$S_{spe-iX}=\gamma_{spe-iX}k_{B}\ln\left(\frac{z-1}{e}\right)\left(\frac{\phi_{i}}{r_{i}}\ln\phi_{i}+\frac{\phi_{X}}{r_{X}}\ln\phi_{X}\right)$
Confinement entropies of nanoparticles	$S_{con-iX}=-k_{B}\frac{\phi_{X}}{r_{X}}\left(\ln\phi_{X}+\tanh\left(\frac{R_{X}}{r_{0,i}}-1\right)\frac{4\phi_{X}-3\phi_{X}^{2}}{\phi_{i}}\right)$
